# *Lycium barbarum* Polysaccharides Regulating miR-181/Bcl-2 Decreased Autophagy of Retinal Pigment Epithelium with Oxidative Stress

**DOI:** 10.1155/2023/9554457

**Published:** 2023-01-05

**Authors:** Yi-Jing Yang, Ying Wang, Ying Deng, Xiao-Qing Liu, Jing Lu, Jun Peng, Jie Li, Ya-Sha Zhou, Hui-An Zhu, Bo Li, Yu-Hui Qin, Qing-Hua Peng

**Affiliations:** ^1^Hunan University of Chinese Medicine, Changsha, 410208 Hunan Province, China; ^2^Institute of Ophthalmology and Otolaryngology of Chinese Medicine, Changsha, 410208 Hunan Province, China; ^3^Hunan Provincial Key Laboratory for the Prevention and Treatment of Ophthalmology and Otolaryngology Diseases with Chinese Medicine, Changsha, 410208 Hunan Province, China; ^4^Department of Ophthalmology, The First Affiliated Hospital of Hunan University of Chinese Medicine, Changsha, 410007 Hunan Province, China

## Abstract

Disturbed structure and dysfunction of the retinal pigment epithelium (RPE) lead to degenerative diseases of the retina. Excessive accumulation of reactive oxygen species (ROS) in the RPE is thought to play an important role in RPE dysfunction and degeneration. Autophagy is a generally low-activity degradation process of cellular components that increases significantly when high levels of oxidative stress are present. Agents with antioxidant properties may decrease autophagy and provide protection against RPE dysfunction and damage caused by ROS. *Lycium barbarum* polysaccharide (LBP) has been widely studied as an antioxidant and cell-protective agent. Therefore, we designed this study to investigate the effects of LBP, which inhibits miR-181, on autophagy in retinal pigment epithelium (RPE) with oxidative stress in vitro and in vivo. In the current study, we found that the highly expressed miR-181 downregulated the expression of Bcl-2 in hydrogen peroxide- (H_2_O_2_-) induced ARPE-19 cells, resulting in an increase in ROS, apoptosis, and autophagy flux. LBP inhibited the expression of miR-181, decreased the levels of ROS, apoptosis, and autophagy flux, and increased cell viability in H_2_O_2_-induced ARPE-19 cells, suggesting that LBP provides protection against oxidative damage in ARPE-19 cells. We also found that LBP decreased RPE atrophy and autophagy flux in rd10 mice. Taken together, the results showed that LBP has a protective effect for RPE under oxidative stress by inhibiting miR-181 and affecting the Bcl-2/Beclin1 autophagy signaling pathway.

## 1. Introduction

The retinal pigment epithelium (RPE) is a monolayer of pigmented cells arranged in the outermost layer of the retina [1]. The RPE sheet is an important structure that connects the outer Bruch's membrane and choroid to the inner photoreceptor cells [2]. There are microvilli structures extending between the outer photoreceptor segments inside the RPE cells, which are involved in phagocytosis of the RPE [2, 3]. The single-layered RPE forms a choroid-blood-retinal barrier with Bruch's membrane and the choroid outside the retina, which controls the transport of material by retinal neurons. The RPE contains melanin, which can reduce ultraviolet light damage to the retina and inner nerves. In addition, the RPE is critical for visual function and photoreceptor homeostasis, because RPE phagocytosis recycles photoreceptor outer segments and the complex metabolic system of the RPE reduces excessive accumulation of reactive oxygen species (ROS) [3–5].

Disrupted structure and dysfunction of the RPE can lead to retinal degenerative diseases, such as retinitis pigmentosa (RP), age-related macular degeneration (AMD), and Stargardt's disease (STGD), in which RPE dysfunction and damage are involved in the development [6]. Therefore, understanding the role of RPE in retinal degeneration and delaying the dysfunction and damage of RPE cells could effectively alleviate retinal degeneration (RD). There is increasing evidence that oxidative stress plays a critical role in RPE dysfunction and damage [7, 8]. Exposure of the retina to ROS results in altered expression of microRNAs (miRNAs) as well as altered expression of genes that may be involved in the pathogenesis and progression of RD [9]. Previous studies have shown that microRNA-181 (miR-181), as a posttranscriptional regulatory molecule, has taken part in the injury of RPE cells by oxidative stress [10].

Autophagy is a degradation process mediated by lysosomes for damaged cellular components, including those damaged by ROS [11]. In this way, the metabolic needs of the cell itself and the renewal of certain organelles are satisfied. Autophagy is a defense and stress regulatory mechanism and acts in the development of immunity, infections, inflammation, tumors, cardiovascular diseases, and neurodegenerative diseases of the body [12–14]. In the state of oxidative stress, mitochondrial ROS homeostasis is disturbed, and its excess accumulation can contribute to membrane lipid peroxidation and structural damage to organelles and also stimulate autophagy to eliminate the damaged sites [15]. Studies have found that autophagy plays an important role in the alleviation of oxidative stress by exogenous antioxidants [16, 17]. Most antioxidants alleviate oxidative stress and reduce apoptosis by regulating the autophagy signaling pathway. Beclin1 has been reported to be a key factor in autophagy formation by regulating the lipid kinase activity of VPS34 (vacuolar protein-sorting 34); Beclin1 is also a multifunctional protein with a BH3 domain that can interact with antiapoptotic protein B-cell lymphoma/leukemia-2 (Bcl-2) and plays a dual role in regulating autophagy and apoptosis [18].

*Lycium barbarum* polysaccharide (LBP) is the active ingredient extracted from the ripe fruit of *Lycium barbarum* and performs a vital function in the antiaging and antioxidant function of wolfberry [19]. The effects of LBP on antioxidants and cell protection have been extensively researched. LBP can effectively resist free radical peroxidation and maintain mitochondrial function [20, 21]. LBP has exhibited high efficacy against oxidative damage and cell apoptosis in hydrogen peroxide- (H_2_O_2_-) induced ARPE-19 cells [22]. In this study, we investigated the effects of LBP, which inhibits miR-181, on autophagy in the oxidative stress response of RPE in vitro and vivo.

## 2. Material and Method

### 2.1. Reagents and Chemicals

*Lycium barbarum* polysaccharide powder, 50% purity (P7850, Beijing Solarbio Life Sciences Co., Ltd.), Hematoxylin and Eosin Staining Kit (G1315-500ml, Beijing Solarbio Science & Technology Co., Ltd.), Annexin V-APC Apoptosis Detection Kit (P-CA-207, Procell), CM-H2DCFDA (C6827, Invitrogen), EDTA (324504, Millipore), TRIzol (15596026, Thermo), methylene-bis-acrylamide (146072, Sigma), Tris (T1503, Sigma), glycine (G8790, Sigma), diethyl pyrocarbonate (683520, Sigma), DMEM/F12 (SLM-243-B, Sigma), skimmed milk powder (P1622, Applygen Technologies Inc.), protease inhibitor (P1265, Applygen Technologies Inc.), protein phosphatase inhibitor (P1260, Applygen Technologies Inc.), 6X sample buffer (CW0610, Cowin Bio), RIPA lysis buffer (HY-K1001, MedChemExpress), CCK8 assay kit (ab228554, abcam), mRNA reverse transcription kit (CW2569, Cowin Bio), miRNA reverse transcription kit (CW2141, Cowin Bio), UltraSYBR Mixture (CW2601, Cowin Bio), Bcl-2 antibody (12789-1-AP, Proteintech), Beclin1 antibody (bs-1353R, BiOSS), LC3A/B antibody (bs-8878R, BiOSS), Atg5 antibody (10181-2-AP, Proteintech), *β*-actin antibody (66009-1-Ig, Proteintech), HRP goat anti-mouse IgG (SA00001-1, Proteintech), HRP goat anti-rabbit IgG (SA00001-2, Proteintech), miR-181a mimic, miR-181a inhibitor, and noncoding RNA (NC) were purchased from Shanghai Jikai Gene.

### 2.2. Preparation of LBP Solution

The impurity of LBP powder mainly contains betaine, zeaxanthine, physalein, carotene, riboflavin, niacin, vitamin C, and so on. The powder was dissolved in distilled water at a concentration of 160 mg/L. The dissolved LBP was filtered using a mixed cellulose ester membrane with a pore size of 0.22 *μ*m (ROJB91514, MF-Millipore). After dilution of the solution, the effects of different concentrations of solubilized LBP on the survival rate of ARPE-19 cells were detected by CCK8.

### 2.3. Plasmid Construction and Cell Transfection

The wild-type pcDNA-Bcl2-EGFP (wt pBcl-2) and mutant pcDNA-Bcl2-EGFP (mut pBcl-2) plasmids were constructed by Shanghai Jikai Gene. GAATGT is the binding site of Bcl-2 and miR-181a, as well as the mutation site in the mut pBcl-2 (Table 1).

The ARPE-19 cell line was purchased from Jennio Biotech Co., Ltd. (Guangzhou, China) and was cultured in a 24-well plate (702011, NEST) at a concentration of 1 × 10^5^ cells/mL for 24 h. When cell confluence reached about 80%, small RNAs (miR-181a mimic and NC) and plasmids (wt pBcl-2 and mut pBcl-2) were transfected, and luciferase activity was detected 24 h later. To further test whether miR-181a can directly regulate the expression of Bcl-2 in ARPE-19 cells, we used RT-qPCR and Western blotting to detect the mRNA and protein expression of Bcl-2 while the miR-181a mimic or NC and wt pBcl-2 or mut pBcl-2 after transfection. Each group repeated three-well plates.

### 2.4. ARPE-19 Cell Culture and Treatment

ARPE-19 cells were cultured in a 1 : 1 mixture of Dulbecco's Modified Eagle Medium and Ham F12 (DMEM/F12) containing 10% fetal bovine serum (FBS) and 100 U/mL penicillin and 0.1 mg/mL streptomycin (both Invitrogen), at 37°C in 5% CO_2_, and cells were passaged three times a week. The cells damaged by oxidative stress were maintained by incubating the ARPE-19 cells with 50 *μ*L (concentration of 200 *μ*mol/L) hydrogen peroxide (H_2_O_2_), and the details and results were described in our previous study (Network pharmacology-based identification of key targets of Ziyin Mingmu Pills acting on age-related macular degeneration, submitted to Evidence-Based Complementary and Alternative Medicine). RNA transfection reagent EntransterTM-R4000 (4000-4, Engreen Biosystem) was used to transfect miR-181 mimic or miR-181 inhibitor into ARPE-19 cells damaged by oxidative stress. The routinely cultured ARPE-19 cells were set as the normal group.

### 2.5. Flow Cytometry Tests the Apoptosis Rate and ROS Content

ARPE-19 cells were divided into the normal group (*n* = 6), cultured as usual; the model group (*n* = 6), treated with H_2_O_2_ and cultured as usual; the LBP low-dose group (LBP-LD, *n* = 6), the model group cultured as usual and treated with 50 *μ*L 10 mg/L LBP solution; the LBP medium-dose group (LBP-MD, *n* = 6), the model group cultured as usual and treated with 50 *μ*L 20 mg/L LBP solution; and the LBP high-dose group (LBP-HD, *n* = 6), the model group cultured as usual and treated with 50 *μ*L 40 mg/L LBP solution. All cells were cultured in 24-well plates for 24 hours.

Digest the adherent ARPE-19 cells with trypsin (HY-P71773, MedChemExpress), take 5 × 10^5^ cells/mL after cell counting, centrifuge at 1000 rpm at 4°C for 10 minutes, and discard the supernatant. Add 1 mL of phosphate-buffered saline (PBS, P1010, Solarbio), shake gently to suspend the cells, then centrifuge at 1000 rpm for 10 min at 4°C, and discard the supernatant. Suspend the cells in 200 *μ*L of binding buffer, add 10 *μ*L of Annexin V-APC, mix gently, and allow to react at room temperature in the dark for 15 minutes. Add 300 *μ*L binding buffer (total reaction volume of 500 *μ*L) and 5 *μ*L of propidium iodide stain and test the apoptosis rate within 1 hour by flow cytometry.

Treated cells were collected by digestion, washed once with PBS buffer, and then resuspended in 1 mL of PBS buffer. The fluorescent probe CM-H2DCFDA was added at a final concentration of 1 M/mL and incubated at 37°C for 30 minutes, and the accumulation of ROS in live cells was analyzed by flow cytometry.

### 2.6. Quantitative Real-Time Polymerase Chain Reaction (RT-qPCR) Assay

After treatment, ARPE-19 cells were washed twice with PBS and TRIzol (Thermo) was added to extract total RNA. RNA concentration (ng/*μ*L) = A260^∗^dilution factor^∗^40; the concentration is 100 ng/*μ*L-200 ng/*μ*L; RNA purity = OD260/OD280; the ratio is qualified from 1.8 to 2.0. mRNA was transcribed into cDNA using a reverse transcription kit (CW2569, Cowin Bio). The miRNA was transcribed using the miRNA reverse transcription kit (CW2141, Cowin Bio). RT-qPCR amplification was performed using SYBR. Primers designed in this study are listed in Table 2.

### 2.7. Immunofluorescence

After treatment of ARPE-19 monolayer cells, cells were fixed in 4% PFA for 30 minutes at room temperature, washed twice with PBS for 3 minutes, and treated for 2 minutes on 0.1% TX-100 (X100, Supelco) at room temperature to permeabilize the cell membrane. Add 30 *μ*L of the diluted primary antibody in PBS with 1% BSA/TBS to the parafilm, incubate the cells for 30 minutes at room temperature, and then wash three times with PBS for 3 minutes. The procedure for incubating the secondary antibody is the same as that for the primary antibody. Finally, add 10 *μ*L of mounting medium (03989, Sigma-Aldrich), let it stand at 37°C for 30 minutes, and then you can observe it with the Leica DM4 B fluorescence microscope.

### 2.8. Protein Extraction and Western Blot Assay In Vitro

Wash the treated ARPE-19 cells at 4°C with PBS and then add RIPA lysis buffer and isolate the protein. Follow the instructions of the BCA protein quantification kit to measure the protein concentration. Depending on the protein quantification result, transfer the sample to the SDS-PAGE gel (P1200, Solarbio) and start electrophoresis at 75 V until the bromophenol blue reaches the bottom. The transferred membranes were saturated with 5% skim milk in PBS for 2 hours. The primary antibodies (Table 3) were incubated for 12 hours at 4°C with a shaker, and the secondary antibodies (Table 4) were incubated for 90 minutes at room temperature with a shaker. The membrane was slid onto ECL chemiluminescence reagent and incubated for 1 minute. The film was exposed and developed in the darkroom. Quantity One software was used to analyze the grayscale of the images.

### 2.9. Animals

Retinal degeneration in rd10 mice begins 16 days after birth, has a late onset, and progresses slowly, so it is often used as a model for RD research [23]. The experimental protocols were approved by the Animal Ethics Committee of Hunan University of Chinese Medicine. The ethics approval number is LL2021042605. The rd10 mice were purchased from Jackson Laboratory (USA), and the certificate number is 1911A11353. C57/BL6 wild-type mice were purchased from Hunan Slake Jingda Experimental Animal Co, Ltd. (China). The certificate number is 43004700062254.

### 2.10. Drug Administration

Forty 4-week-old rd10 mice were randomly divided into 5 groups, including the model group (*n* = 8), the LBP low-dose group (LBP-LD, *n* = 8), the LBP high-dose group (LBP-HD, *n* = 8), the miR-181 mimic group (*n* = 8), and the miR-181 inhibitor group (*n* = 8). All C57/BL6 mice were assigned to the control group (*n* = 8). The model group and the control group were administered 0.9% saline orally. The LBP-LD group and the LBP-HD group received oral doses of 3.6 g/kg and 7.2 g/kg per day, respectively. The miR-181 mimic group and the miR-181 inhibitor group received a local injection into the vitreous at a dose of 0.5 *μ*g per eye every four days using a microsyringe (Hamilton, 700 syringe and 33 G needle). Mice were fed at a temperature of approximately 22°C, and oral administration lasted 28 days (a course of treatment with the Chinese medicine *Lycium barbarum*).

### 2.11. Electroretinography Examination

After oral administration was completed, mice were treated with tribromoethanol (1329-86-8, Hefei TNJ Chemical Industry Co., Ltd.) for anesthesia and treated with 0.5% tropicamide for mydriasis. Electroretinography was performed using the Micron IV system (Phoenix Research Labs, USA).

### 2.12. Histopathological Examination of Retinal Tissues

After the ERG examination was completed, the mice were killed and the eyeballs were harvested. The eyeballs were fixed with eyeball fixed liquid (B0006, Powerful Biology). The fixed tissue was sequentially dehydrated, dipped in wax, embedded, and sectioned. HE staining was performed according to the instructions of the staining kit for histopathological examination (HE) (C0105S, Beyotime Biotechnology). Images were captured using a Leica DCM8 microscope.

### 2.13. Western Blot Assay In Vivo

The retina was collected from the mice to perform a Western blot test. The process, procedures, and reagents are described in Section 2.5.

### 2.14. Statistical Analysis

Statistical analyses were performed using SPSS version 25.0. All data was expressed as the mean ± standard deviation (SD). For comparison of quantitative data between two groups, the normality test was performed first. If all groups accorded with normality and the variance between the two groups was equal, *t*-test was used for comparison between groups. Otherwise, take into account the nonparametric Wilcoxon rank-sum test. For comparison between multiple groups, one-way analysis of variance (one-way ANOVA) was used for comparison between groups while the continuous data followed the normal distribution and homogeneity of variance, and the Bonferroni method was further used for the pairwise comparison as the difference between groups was statistically significant. If there was no normal distribution or unequal variance, the Kruskal-Wallis rank-sum test was used for comparison among multiple groups. When there are overall statistical differences between groups, DSCF method is further used for multiple comparisons. *p* < 0.05 was considered statistically significant.

## 3. Results

### 3.1. The Cell Viability of LBP Detected by CCK8

**Figure 1:**
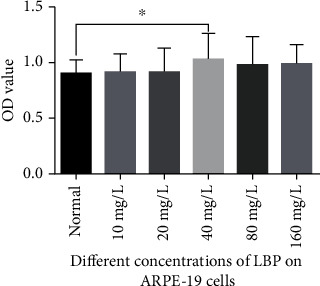
Results of ARPE-19 cell viability with various concentrations of LBP. Quantitative analysis of data is presented as the mean ± SD, ^∗^*p* < 0.05.

We first examined the effect of LBP on the viability of ARPE-19 cells, which are commonly used in RD cell experiments. The results of CCK8 assay showed that the concentration of LBP in dissolved form at 40 mg/L had the best effect on the survival rate of ARPE-19 cells (Figure 1).

### 3.2. miR-181 Had a Significant Regulatory Effect on Bcl-2

**Figure 2:**
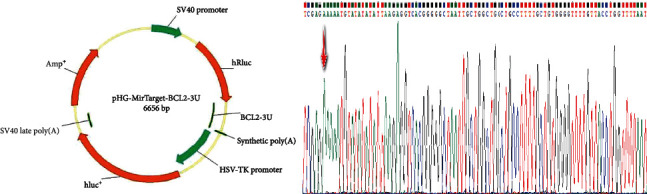
pHG-MirTarget-BCL2-3U plasmid maps and the Sanger sequence of pHG-MirTarget-Bcl2-3U plasmid map. The red arrow indicates the starting position of Bcl-2.

**Figure 3:**
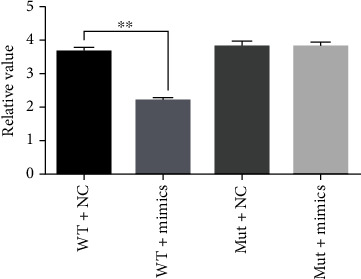
Relative level of dual-luciferase. WT+NC represents pHG-MirTarget-BCL2-3U and no coding miRNA; WT+mimics represent pHG-MirTarget-BCL2-3U and miR-181 mimic; mut+NC represents pHG-MutTarget-BCL2-3U and no coding miRNA; mut+mimics represent pHG-MutTarget-BCL2-3U and miR-181 mimic. Two-sided *t*-test was performed between other groups and the WT and NC groups, ^∗∗^*p* < 0.01.

We successfully constructed the Bcl-2 plasmid and verified it by sequencing (Figure 2). The results of the dual luciferase assay illustrated that hsa-miR-181a-5p has significant regulatory effect on Bcl-2 gene (Figure 3). The WT+NC group represents ARPE-19 cells transfected with pHG-MirTarget-BCL2-3U plasmid and no coding miRNA (final concentration 50 nM). The WT+mimic group stands for ARPE-19 cells transfected with pHG-MirTarget-BCL2-3U plasmid and miR-181 mimic (final concentration 50 nM). The mut+NC group represents ARPE-19 cells transfected with the pHG-MutTarget-BCL2-3U plasmid and no coding miRNA (final concentration 50 nM). The mut+mimic group represents ARPE-19 cells transfected with pHG-MutTarget-BCL2-3U plasmid and miR-181 mimic (final concentration 50 nM).

**Figure 4:**
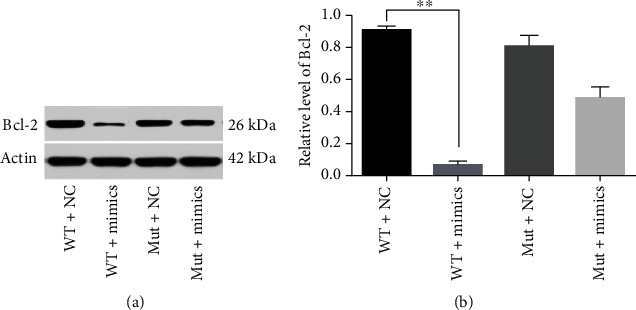
The effect of hsa-miR-181a-5p on the expression of Bcl-2 protein bands (a) and the results of grayscale statistical analysis (b), using two-sided *t*-test, ^∗∗^*p* < 0.01.

Western blot assay was performed to determine the regulatory effect of hsa-miR-181a-5p on human Bcl-2 gene. The results showed that hsa-miR-181a-5p has a significant regulatory effect on the Bcl-2 gene (Figure 4).

### 3.3. LBP Decreased the Generation of Excessive ROS in ARPE-19

**Figure 5:**
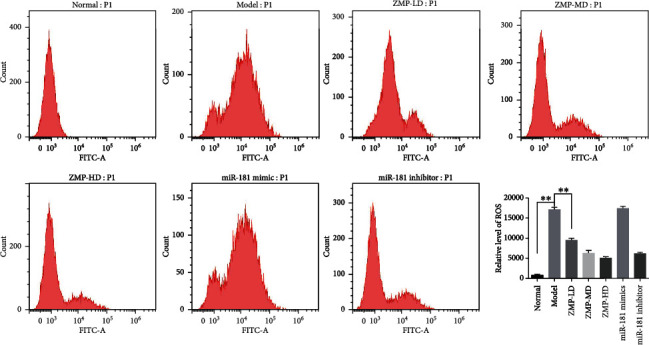
LBP inhibited ROS levels in hydrogen peroxide-induced ARPE-19 cells. Relative levels of ROS in each group of ARPE-19 cell were measured by flow cytometry. Quantitative analysis of data is presented as the mean ± SD, ^∗∗^*p* < 0.01.

The generation of excessive ROS is the main factor leading to oxidative stress in the RPE, and oxidative stress is considered to be one of the main causes of RPE cell damage and apoptosis. Flow cytometry results found that hydrogen peroxide led to a remarkable increase in cellular ROS in ARPE-19 cells (Figure 5). Inhibition of miR-181 contributes to a decrease in cellular ROS levels, while LBP treatments cause the same change in ARPE-19 cells. Comparison between LBP-LD and LBP-HD groups showed that with the increase of LBP dose, the amount of ROS in cells decreased significantly. Possibly, LBP decreases the formation of excessive ROS in ARPE-19 cells in a dose-dependent manner.

### 3.4. LBP Protected ARPE-19 from Apoptosis

**Figure 6:**
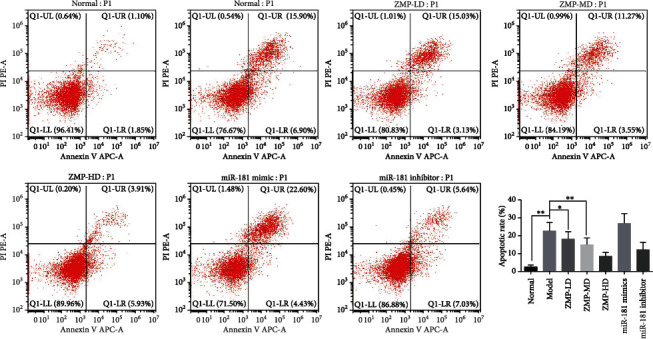
LBP attenuated the apoptosis induced by hydrogen peroxide in ARPE-19 cells. Apoptotic rate in each group of ARPE-19 cell was measured by flow cytometry. Quantitative analysis of data is presented as the mean ± SD, ^∗^*p* < 0.05 and ^∗∗^*p* < 0.01.

To evaluate the protective effect of LBP on hydrogen peroxide-induced ARPE-19 cells, apoptosis detection was carried out by flow cytometry. The results of the Annexin V-FITC assay showed that the apoptosis rate of ARPE-19 cells induced by hydrogen peroxide increased significantly (see Figure 6). Inhibition of miR-181 leads to a decrease in the apoptotic rate of ARPE-19 cells with oxidative stress, and LBP treatments induce the same change in ARPE-19 cells with excessive ROS accumulation. Comparison between the LBP-LD and LBP-MD groups showed that the apoptotic rate of cells decreased significantly with increasing LBP dose.

### 3.5. LBP Inhibited miR-181 and Affected the mRNA Expression of Bcl-2/Beclin1 Signaling

**Figure 7:**
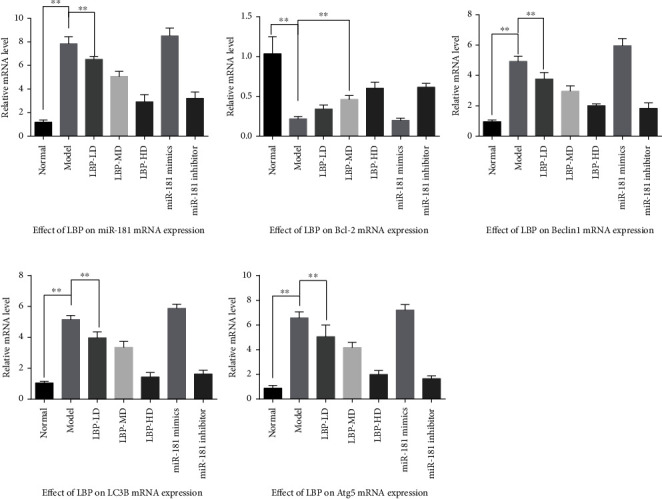
The effect of LBP on mRNA expression of miR-181a-5p, Bcl-2, Beclin1, LC3, and Atg5 in oxidative stress ARPE-19 cell. Values are presented as the mean ± SD, ^∗^*p* < 0.05 and ^∗∗^*p* < 0.01.

The QPCR assay was used to determine the effect of LBP on mRNA in ARPE-19 cells with oxidative stress. The results revealed that the relative level of miR-181a-5p, Bcl-2, Beclin1, LC3B, and Atg5 mRNA in hydrogen peroxide induced oxidative stress ARPE-19 groups. The results indicated that mRNA expression of miR-181 scaled-up, mRNA expression of Bcl-2 drop down, and mRNA expression of autophagy genes including Beclin1, LC3B, and Atg5 increased in ARPE-19 cells with oxidative stress; inhibition of miR-181 promoted mRNA expression of Bcl-2 and restrained the mRNA expression of autophagy gene Beclin1, LC3B, and Atg5 in ARPE-19 cell with oxidative stress; LBP-HD and LBP-MD may have the same effect as miR-181 inhibitor (Figure 7).

### 3.6. LBP Improved the Cell Viability in Oxidative Stress ARPE-19 Cells

**Figure 8:**
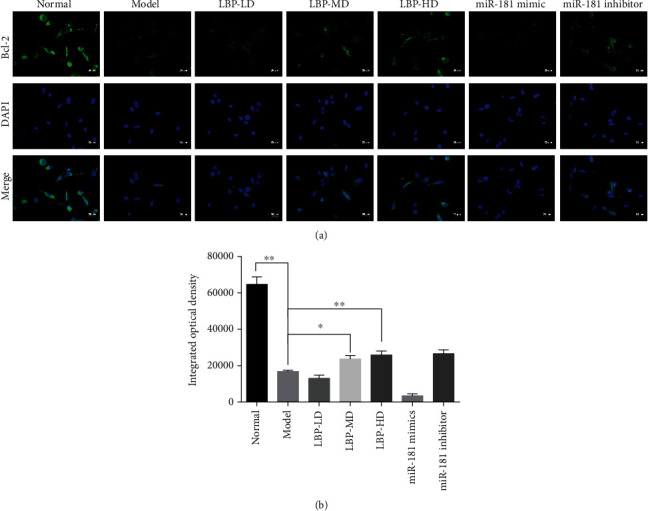
LBP increased the expression of Bcl-2 in oxidative stress ARPE-19 cells. (a) Cells were transfected with fluorescent-tagged Bcl-2 reporter and treated with different drugs according to their grouping. Images were taken by Leica fluorescence microscope (scale bars, 25 *μ*m). (b) The expression of Bcl-2 was assessed by integrated optical density, data are presented as the mean ± SD, ^∗^*p* < 0.05 and ^∗∗^*p* < 0.01.

Following the findings of QPCR and flow cytometry in hydrogen peroxide-pretreated ARPE-19 cells, we further employed immunofluorescence assay to evaluate the protein expression of Bcl-2 in oxidative stress ARPE-19 cells, which implies the ARPE-19 cell viability with oxidative damage. Images were taken by Leica fluorescence microscope (scale bars, 25 *μ*m, Figure 8). The integrated optical density (IOD) displayed that hydrogen peroxide decreased expression in ARPE-19 cells; inhibition miR-181 increased expression in ARPE-19 cells. With sufficient dose of LBP (LBP-HD and LBP-MD), the expression of Bcl-2 has a tendency to increase, and there is no difference in the expression between LBP-HD and miR-181 inhibitor.

### 3.7. Beclin1 Signaling Was Involved in LBP Regulating miR-181/Bcl-2-Protected Oxidative Stress ARPE-19

**Figure 9:**
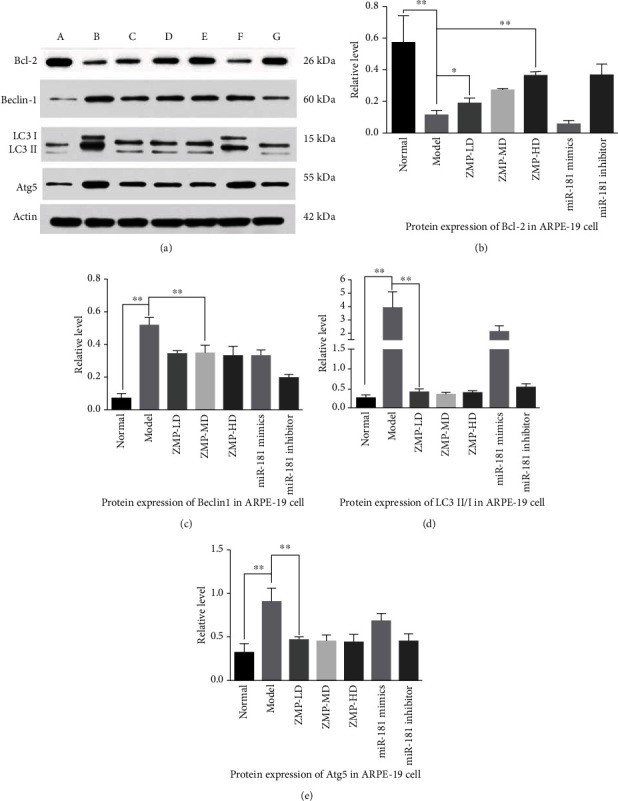
Effects of LBP extract on the protein expression of Bcl-2, Beclin1, LC3B, and Atg5 in ARPE-19 cells. (a) A represents the normal group; B represents the model group; C represents the LBP-LD group; D represents the LBP-MD group; E represents the LBP-HD group; F represents the miR-181 mimic group; G represents the miR-181 inhibitor group. (b–e) The protein expression relative level of Bcl-2, Beclin1, LC3B, and Atg5 in ARPE-19 cells; the data appeared as the mean ± SD, ^∗^*p* < 0.05 and ^∗∗^*p* < 0.01.

In order to explore whether autophagy is involved in LBP-mediated protection against hydrogen peroxide-induced oxidative stress ARPE-19 cells, Western blot was employed to investigate the protein expression of Bcl-2, Beclin1, LC3BII/I, and Atg5 in ARPE-19 groups (Figure 9). Compared with the normal group, the relative level of Bcl-2 protein was significantly decreased in the model group. The relative level of Bcl-2 protein in all LBP group was significantly increased compared with the model group. The results suggest that the protein expression of Bcl-2 decreased and autophagy protein including Beclin1, LC3B, and Atg5 increased expression in ARPE-19 cell with oxidative stress; inhibition of miR-181 increased the protein expression of Bcl-2 and decreased protein expression of Beclin1, LC3B, and Atg5, and LBP-MD and LBP-HD can play the same effect as miR-181 inhibitor.

### 3.8. ERG Analysis

**Figure 10:**
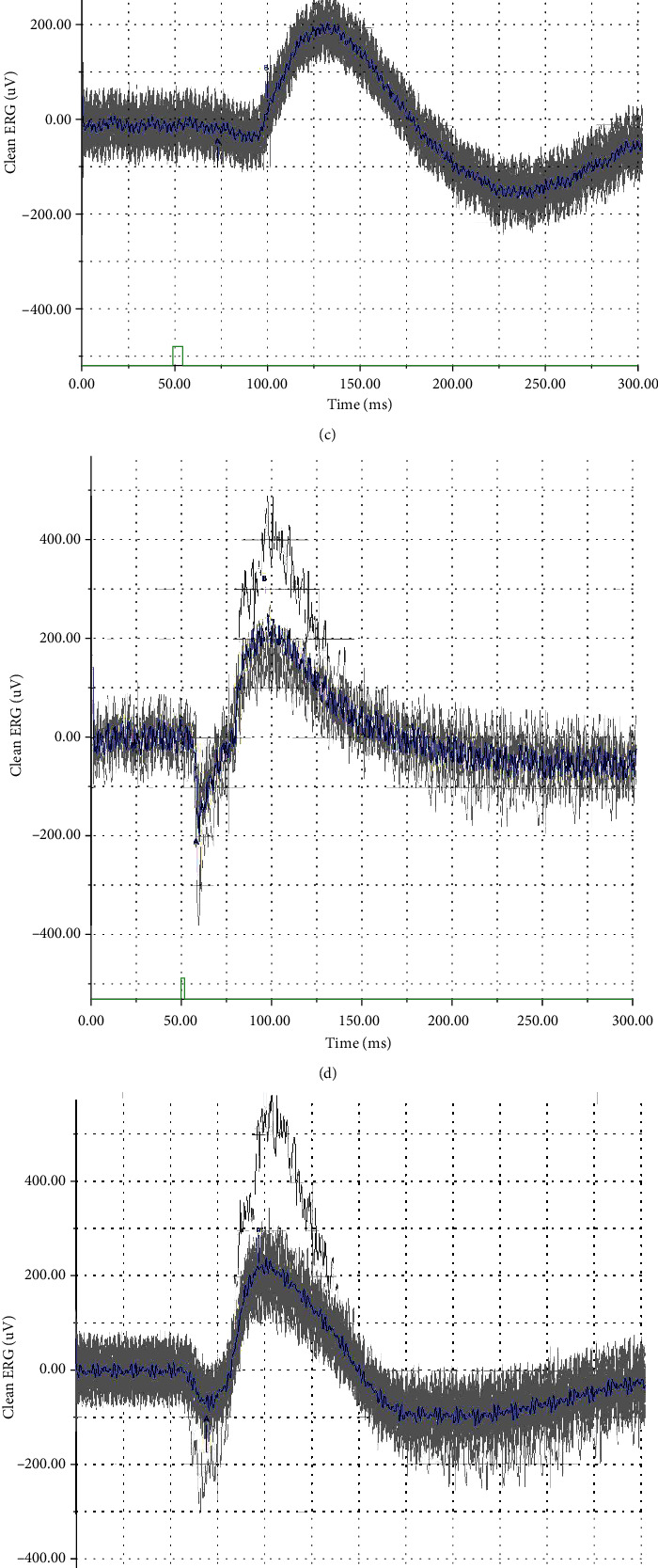
The effect of FSE extract on the visual function of mice detected by ERG. (a) ERG responses of C57 mice in the normal group; (b) ERG responses of rd10 mice in the model group; (c) ERG responses of rd10 mice in the LBP-LD group; (d) ERG responses of rd10 mice in the LBP-HD group; (e) ERG responses of rd10 mice in the miR181 mimic group; (f) ERG responses of rd10 mice in the miR181 inhibitor group; a-wave (g) and b-wave (h) exhibit in retinal tissues of different experimental groups. Data is presented as the mean ± SD, ^∗^*p* < 0.05 and ^∗∗^*p* < 0.01.

The amplitude wave of electroretinogram depends on the function of RPE, photoreceptors, bipolar cells, Müller cells and retinal choroidal blood circulation. Involvement of RPE generally leads to abnormal ERG in both a-wave and b-wave. ERG analysis was used to observe the changes in the visual function of mice in all the groups (Figure 10). The figures displayed that the amplitude of the a-wave and b-wave was recorded in all the groups. The amplitude of a-wave and b-wave in the mice model group both was at low levels, and inhibition miR-181 markedly increased the amplitude of a-wave and b-wave. LBP-HD works the same as inhibitor miR-181. The results suggested LBP and inhibition miR-181 has a beneficial impact on the visual function of rd10 mice.

### 3.9. Effect of LBP on RPE Changes in rd10 Mice

**Figure 11:**
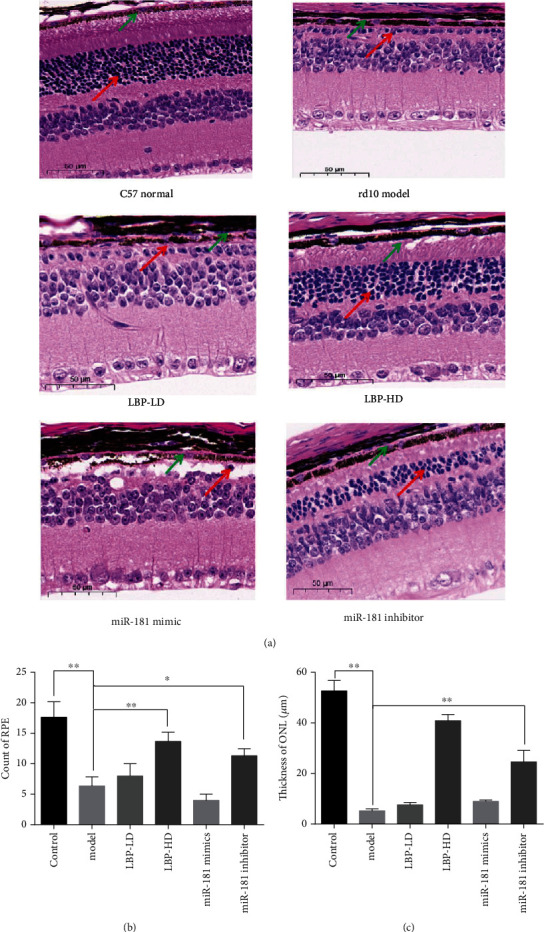
The effect of LBP on retina changes in rd10 mice detected by HE staining. (a) Retina HE staining in each group; green arrows indicate RPE layers and red arrows indicate outer nuclear layer (ONL) which comprise of cones and rods, scale bars, 50 *μ*m; (b) ONL thickness changes in different mice groups; (c) the count of RPE in each groups. Data is presented as the mean ± SD, ^∗∗^*p* < 0.01.

HE staining was carried out to observe the RPE changes in rd10 mice retinal tissue, C57 mice as the normal group. As shown in Figure 11, the structure of retinal tissue in the model group (rd10 mice) had a rupture or disappearance of the RPE layer (green arrow) and loss of a large number of photoreceptor cells (red arrow). The miR-181 mimic group has the identical changes of morphology to the model group. In comparison to the model group, the overall retina in the LBP group was well structured. There may be a dose-dependent manner of LBP in the survival of RPE cells and the retinal structural arrangement. The miR-181 inhibitor group has the same outcome as the LBP-HD group. In addition, LBP and inhibition miR-181 could effectively protect photoreceptor from death.

### 3.10. Effect of LBP Regulating miR-181/Bcl-2 on Beclin1 Signaling in rd10 Mice Retina

**Figure 12:**
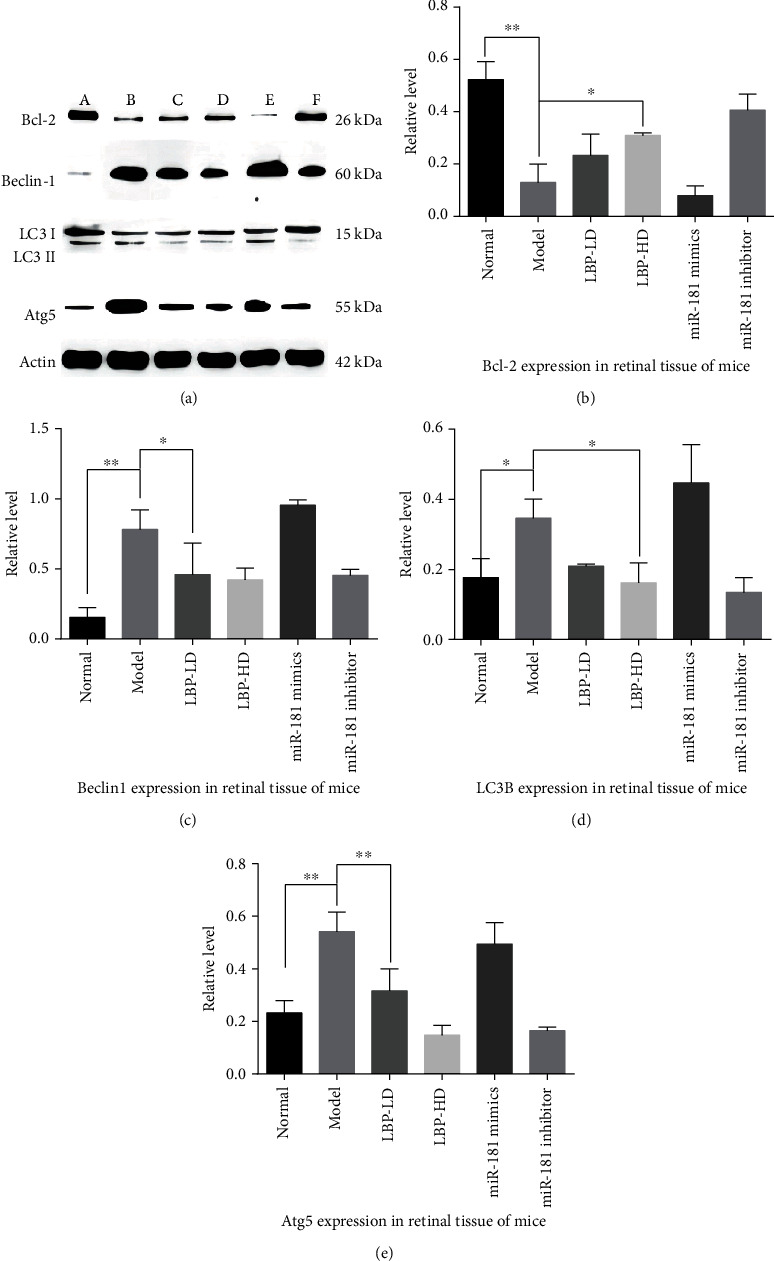
The effects of LBP extract on the protein expression of Bcl-2/Beclin1 signaling in rd10 mice retina. (a) A represents the normal group; B represents the model group; C represents the LBP-LD group; D represents the LBP-HD group; E represents the miR-181 mimic group; F represents the miR-181 inhibitor group. (b–e) The protein expression relative level of Bcl-2, Beclin1, LC3B, and Atg5 in rd10 mice retina; the data appeared as the mean ± SD, ^∗^*p* < 0.05 and ^∗∗^*p* < 0.01.

WB assay revealed the effects of LBP on the protein expression of Bcl-2, Beclin1, LC3B, and Atg5 in retinal tissue of mice groups (Figure 12). Compared with the normal group, the Bcl-2 expression was significantly decreased, and the autophagy proteins including Beclin1, LC3B, and Atg5 were increased in the model group. Inhibition of miR-181 and the expression of Bcl-2 aggravated, while the expression of Beclin1, LC3B, and Atg5 attenuated; the expressions of Bcl-2/Beclin1 signaling in the LBP-HD group have a similar effect to miR-181 inhibitor. By comparison, the protein expression of the LBP-LD group to the LBP-HD group indicated that there was a dose-dependent relationship in LBP regulating miR-181/Bcl-2 on Beclin signal pathway.

## 4. Discussion

The understanding of RPE demonstrates that oxidative damage is one of the main causes of retinal degeneration [3, 24]. The retina is a tissue with high oxygen consumption and high oxidation products. Hydrogen peroxide is produced by the RPE as part of cellular oxidative metabolism, which provides several mechanisms to support normal photoreceptor function [25]. Excessive accumulation of ROS in the retina and diminished antioxidant capacity in the cells leads to RPE cell dysfunction and damage [24, 26]. Besides, exposure of the RPE to ROS-altered expression of miRNA may be involved in the pathogenesis and progression of RD [27]. The ARPE-19 cell line used in this study was isolated from primary human RPE cells and is commonly used as an in vitro model of RD [28]; rd10 mice have a missense mutation in phosphodiesterase 6B, and these mice have retinal degeneration that complete at 60 days of age [29]. Our study showed that miR-181 significantly increased in the oxidative stress response of ARPE-19 cells and was associated with an increase in oxidative stress and autophagy, whereas antioxidants and inhibition of miR-181 decreased oxidative stress and autophagy flux. Moreover, injection of antioxidants and miR-181 inhibitors ameliorated RPE injury in rd10 mice. Agents with antioxidant properties could rebalance miRNA expression and provide protection against RPE dysfunction and damage induced by ROS.

Previously, miR-181 has been reported to be closely related to the degeneration of visual impairment. miR-181 was highly expressed in the lens tissue of age-related cataract and negatively regulates Bcl-2, thereby inhibiting the proliferation of lens epithelial cells [30]. Recently, a study found that miR-181a is involved in the process of neurodegenerative complaint, and overexpression of miR-181a can activate autophagy in cells [31]. Moreover, one study found that miR-181 is involved in the oxidative stress response of RPE cells as a posttranscriptional regulatory molecule [10]. The Bcl-2 gene is regulated by miR-181, which is also known as the most important regulatory gene in apoptosis and can mediate the release of Beclin1 and other substances by mitochondrial oxidative stress [32, 33]. Our findings hint that oxidative stress-induced miR-181 inhibits Bcl-2 and affects Beclin1 autophagy signaling in the RPE oxidative stress response. Antioxidant regulation of miR-181/Bcl-2 attenuated Beclin1 autophagy signaling and resulted in a protective effect on oxidative stress in the RPE.

Autophagy in the RPE is responsible for retinal homeostasis, and any defect in autophagy disrupts RPE function and triggers retinal degeneration diseases [34]. The Beclin1 gene, an autophagy-related gene, is in antagonism to the Bcl-2 gene to regulate autophagy [35]. Studies have demonstrated that oxidative stress-induced damage to proteins and organelles can cause Beclin1 to separate from Bcl-2 and form a Beclin1-VPS34 complex, initiating autophagy to eliminate the damaged site [15]. Oxidative stress that decreased the expression of Bcl-2 also abrogates mitochondrial function and exacerbates the accumulation of ROS [36]. Prolonged or severe oxidative stress leads to damage of organelles such as mitochondria, resulting in expansion of autophagy flux which in turn leads to abnormal mitochondrial function [37]. Researchers found that suppressing the expression of Atg5 and Beclin1 can prevent cell death because blocking autophagy significantly decreases caspase-3 [38]. At low stress levels, autophagy is already activated and may serve as an important protection against oxidative injuries [39]. Our results suggest that protein expression of Beclin1, LC3B, and Atg5 is at a low level in C57 mice and maintains this justification. When oxidative stress is high, autophagy is overactivated and contributes to cell injury [40, 41]. The protein expression of Beclin1, LC3B, and Atg5 in the model group in vitro and in vivo is consistent with previous studies.

LBP is the active ingredient extracted from the ripe fruit of *Lycium barbarum* and has been widely studied as an antioxidant and cell-protective agent [19]. One study found that LBP exhibited protective effects against neurotoxicity by reversing H_2_O_2_-induced elevated ROS levels, decreased cell viability, and decreased mitochondrial membrane potential, indicating the amelioration of mitochondrial apoptosis [42]. LBP attenuates chemotherapy-induced oxidative stress by enhancing the efficacy of antioxidant enzymes and attenuating the increased concentrations of oxidation products after cyclophosphamide injection [43]. In recent years, some studies have also demonstrated that the cytoprotective effect of LBP is mediated by autophagy. In an optic nerve injury microglia model induced by bipolar pulsed current, LBP attenuated the inhibition of autophagy by electrical stimulation and attenuated apoptosis and oxidative stress [44]. Treatment of hippocampal neurons with LBP during oxygen-glucose deprivation and reperfusion decreased intracellular expression of LC3B and Beclin1, resulting in a decrease in autophagosomes and an increase in p62 protein levels [45]. Moreover, LBP exerts an excellent function in antioxidant activity, increasing cell viability and against apoptosis effects by regulating miRNAs.

In conclusion, this study shows that oxidative stress upregulates miR-181 and autophagy flux in ARPE-19 cells and rd10 mice. The cytoprotective effects of LBP in oxidatively damaged ARPE-19 cells and rd10 mice were associated with antioxidant activity and a reduction in autophagy through regulation of miR-181/Bcl-2. This suggests that RD treatment with LBP theoretically attenuates oxidative damage and autophagy in the RPE.

## Figures and Tables

**Table 1:** The continuous binding fragment exists between miR-181a and Bcl-2.

Bcl-2(wt)	5′	A	C	T	T	T	G	G	A	A	T	G	T	A	3′
								|	|	|	|	|	|		
miR-181a-5p	5′	U	C	G	C	A	A	C	U	U	A	C	A	A	3′
Bcl-2(mut)	5′	A	C	T	T	T	G	U	G	G	A	U	C	A	3′

**Table 2:** The pair of the primers designed in qPCR.

Target	Primers	Product
miR-181a-5p	CGCAACATTCAACGCTGTCGG	—

Bcl-2 https://www.ncbi.nlm.nih.gov/gene/596	Forward: AGCTGCACCTGACGCCCTT	147 bp
	Reverse: ACATCTCCCGGTTGACGCTCT	

Beclin1 https://www.ncbi.nlm.nih.gov/gene/8678	Forward: CATGGAGAACCTCAGCCGAA	172 bp
	Reverse: ACAGCGTTTGTAGTTCTGACAC	

LC3 https://www.ncbi.nlm.nih.gov/gene/81631	Forward: TTCAGGTTCACAAAACCCGC	163 bp
	Reverse: TCTCACACAGCCCGTTTACC	

Atg5 https://www.ncbi.nlm.nih.gov/gene/9474	Forward: AAGATGTGCTTCGAGATGTGT	144 bp
	Reverse: ACTTTGTCAGTTACCAACGTCA	

*β*-Actin https://www.ncbi.nlm.nih.gov/gene/60	Forward: ACCCTGAAGTACCCCATCGAG	224 bp
	Reverse: AGCACAGCCTGGATAGCAAC	

**Table 3:** The primary antibody information.

Target gene	Cat no.	Host	Dilution	Company
BCL-2	12789-1-AP	Rabbit	1 : 2000	Proteintech
Beclin1	bs-1353R	Rabbit	1 : 2000	BiOSS
LC3A/B	bs-8878R	Rabbit	1 : 2000	BiOSS
Atg5	10181-2-AP	Rabbit	1 : 2000	Proteintech
*β*-Actin	66009-1-Ig	Mouse	1 : 5000	Proteintech

**Table 4:** The secondary antibody information.

Target gene	Cat no.	Dilution	Company
HRP goat anti-mouse IgG	SA00001-1	1 : 5000	Proteintech
HRP goat anti-rabbit IgG	SA00001-2	1 : 6000	Proteintech

## Data Availability

The data is available upon request from the author.
